# A Comprehensive Review of Acne Treatments: Unpacking the Chemical Structures and Effective Bioactive Compounds

**DOI:** 10.1002/hsr2.71803

**Published:** 2026-02-03

**Authors:** Mobina Tajdari, Setare Abolghasemi, Elham Khanniri, Maryam Bayanati, Rahil Hakimimofrad, Mohammad Mahboubi‐Rabbani

**Affiliations:** ^1^ Department of Medicinal Chemistry, Faculty of Pharmacy, Tehran Medical Sciences Islamic Azad University Tehran Iran; ^2^ Department of Food Technology Research, National Nutrition and Food Technology Research Institute, Faculty of Nutrition Sciences and Food Technology Shahid Beheshti University of Medical Sciences Tehran Iran; ^3^ Phytochemistry Research Center Shahid Beheshti University of Medical Sciences Tehran Iran; ^4^ Department of Pharmacoeconomics and Pharma Management, School of Pharmacy Shahid Beheshti University of Medical Sciences Tehran Iran

**Keywords:** acne, bioactive compounds, chemical structure, optimum therapy

## Abstract

**Background and Aims:**

Acne vulgaris is a frequent skin disorder, affecting a large part of the population worldwide, and strongly influencing not only the physical but also the mental aspects of health. The choice of therapy for acne vulgaris is a very difficult one because the multifactorial causality of the disease and interindividual variability in response to treatment should be considered. This article aims to provide a comprehensive review of the drugs used in the treatment of acn\e vulgaris, along with their chemical structure.

**Methods:**

A comprehensive search of relevant databases (PubMed, Google Scholar, Web of Science, and Scopus) was conducted using keywords such as “acne vulgaris”, “chemical structure”, “mode of action”, and “structure–activity relationship”. We reviewed articles published up to 2025. The authors reviewed articles that discussed the chemical structure and structure–activity relationships of drugs used in the treatment of acne vulgaris. In addition, articles on acne vulgaris pharmacotherapy were also reviewed.

**Results:**

In this review, all the current modalities for the management of acne vulgaris are presented and grouped according to their chemical nature, including topical applications and systemic administration. In this light, we go further into the mechanism of action of each treatment, its efficacy and safety, and possible side effects. New therapeutic agents targeting the skin microbiome, inflammation, and hormonal imbalance are also a focus. We finally propose one step for choosing an optimum therapy based on the subject's constitution, the severity of post‐acne scarring, and associated comorbidities.

**Conclusion:**

The present review would benefit general physicians in adapting their management to treat zits and pimples for the improvement of their patients, with particular importance given to the knowledge of the chemical structures of various drugs.

**Abbreviations**
9cRA: 9‐*cis*‐retinoic acid
ADP: adapalene
AMPK: adenosine monophosphate‐activated protein kinase
AP: activator protein
ATRA: all‐trans retinoic acid
AV: acne vulgaris
AZA: azelaic acid
BPO: benzoyl peroxide
BTX‐A: botulinum toxin type A
CBD: cannabidiol
CRABPs: cellular retinoic acid‐binding proteins
CRBPs: cytoplasmic retinol‐binding proteins
CXCL8: C‐X‐C motif chemokine ligand 8
*C. acnes*
: *Cutibacterium acnes*
DNA: deoxyribonucleic acid
EGCG: epigallocatechin3‐gallate
HPA: hypothalamic‐pituitary‐adrenal
IGF‐1: insulin‐like growth factor‐1
IL: interleukin
LBPs: ligand‐binding pockets
LTA4H: leukotriene A4 hydrolase
LTB(4): leukotriene B(4)
MRSA: methicillin‐resistant *Staphylococcus aureus*
NF‐κB: nuclear factor‐κB
PI3K: phosphatidylinositide 3 kinase
*P. acnes*
: *Propionibacterium acnes*
RA: retinoic acid
RAR: retinoic acid receptor
ROS: reactive oxygen species
RXR: retinoid X receptors
SEB‐1: sebocyte‐1
SREBP‐1: sterol regulatory element‐binding protein‐1
TEWL: transepidermal water loss
TLRs: toll‐like receptors
TNF‐α: tumor necrosis factor‐alpha
ZnO NPs: zinc oxide nanoparticles

## 1 Introduction

Acne vulgaris (AV) is the most common form of acne [1]. It is a chronic and occasionally inflamed disease of the pilosebaceous unit [2]. AV affects 85% of adolescents and thus usually starts in the years of preadolescence and continues into adult life. It has psychological repercussions, given that it leads to an increased prevalence of mood disorders, psychiatric hospitalizations, absence of fundamental phenomena at school, unemployment, and suicidal tendencies [3]. AV is classified based on the age of the patient, the morphology of lesions (comedonal, inflammatory, mixed, nodulocystic), distribution—the location on face, trunk, or both, and severity of disease—extent, presence or absence of scarring, post‐inflammatory erythema or hyperpigmentation [4]. Acne within this age bracket, which is referred to as adolescent acne, is thus generally considered to fall within the ages of 10–19 years. Individuals developing acne within the age brackets of 10–24 years should be classified as having young acne. The acne that develops after the age of 25 is described as adult acne [5]. Comedonal or non‐inflammatory lesions include open comedones, also known as blackheads, and closed comedones, also referred to as whiteheads. The inflammatory lesions are papules, pustules, nodules, and cysts [1]. Nodulocystic acne is a dermatological disorder that causes substantial destruction of the dermis. This may also cause scarring of this disease on the face, chest, and back [6]. The four primary factors in acne pathogenesis include; (1) increased androgens which drive increased keratinocyte proliferation, increased sebum production, and increased sebaceous gland size; (2) abnormal keratinocyte proliferation and comedone formation; (3) inflammation to the pilosebaceous follicle; and (4) bacterial colonization, primarily by *Cutibacterium acnes* (*C. acnes*), but also involves other species such as *Staphylococcus epidermidis* and *Staphylococcus aureus*, as they contribute to the inflammation and antimicrobial resistance [7, 8, 9, 10]. The complexity of the acne pathogenesis means that treatment involving a combination of products is usually considered best practice [3]. Overall, acne pathogenesis is not a simple process, involving complex interactions of numerous factors, including follicular hyperkeratinization, increased sebum production, hyper‐inflammatory processes, and microbial dysbiosis, primarily *C. acnes* [11]. New perspectives regarding the host immunology related to *C. acnes* and its subsequent pathophysiological role(s) in acne development have recently been shed. Topical retinoids are the preferred agent for treatment and maintenance therapy for patients with mild‐to‐moderate AV; topical retinoids can be used alone or with benzoyl peroxide (BPO) and topical or oral antibiotics, depending on the severity of treatment. Oral antibiotics are also an important agent in the treatment of inflammatory acne that doesn't respond to topical therapy. As an additional comment, it is relevant to say that neither the oral forms nor the topical forms should be prescribed alone. Oral isotretinoin is preferred for the treatment of severe and nodular AV and moderate forms when there is a presence of scars [12, 13]. Although a wide range of therapeutic options is available for acne, several important research gaps remain. Well‐designed randomized controlled trials are still needed to evaluate the efficacy of natural or biologically derived agents in phase III clinical settings. The long‐term safety of nanoparticle‐based topical delivery systems has not yet been fully established, and a deeper understanding of host immunology and *C. acnes* subtypes is required. In addition, stronger comparative evidence is needed to guide personalized treatment strategies. To address these gaps, the present review provides an integrated synthesis of both established and emerging acne therapies, organized according to their chemical class. By combining mechanisms of action, pharmacological characteristics, and clinical relevance into a single framework, this review aims to offer a clearer and more practical foundation for rational and personalized management of AV. This review aims to provide a comprehensive overview of all major acne treatment options categorized by their chemical structure.

## 2 Methods

This review was conducted to summarize the available acne vulgaris treatments with their chemical structures. Refereed literature was obtained through a search of databases, such as PubMed, Google Scholar, Web of Science, and Scopus, from 1990 to 2025. The keywords included: “acne vulgaris”, “treatment of acne”, “pathogenesis of acne”, “topical therapy”, “oral antibiotics”, “hormonal therapy”, “retinoids”, “anti‐inflammatory agents”, “keratolytic agents”, “chemical structure”, “mode of action”, and “structure‐activity relationship”. Only articles published in English were included. The selection included clinical studies (randomized controlled trials, cohort studies) and review articles (systematic reviews, meta‐analyses, and narrative reviews) discussing any of the pathophysiology or treatment of acne. Articles were screened by title and abstract and assessed for relevance and appropriateness of information, with the full text reviewed to ascertain response to research questions.

### 2.1 Overview of Conventional Treatment Options by Their Chemical Structures

This review classifies drugs for acne treatment according to their molecular composition, intending to gain more information on the structure–activity relationship and mechanism of action (Table 1). Such a basis will allow deep insight into how acne treatments have evolved, identify common therapy targets, and pave the way toward the development of newer and more effective therapeutic approaches for this widespread skin disorder [14]. Additionally, a thorough understanding of acne pathogenesis is essential for a more comprehensive investigation of treatment approaches, as discussed in detail in the supplementary file.

**Table 1:** Summary of the introduced chemical structures of acne treatment.

		Conventional treatments							
Classification of drugs	Drugs	Route of administration	Mechanism of action	Acne severity	Recommended concentration	Side effects	Type of study	Evidence strength	References
Retinoids	Tretinoin	Topical	Regulates the keratinization, differentiation, maturation, and proliferation of keratinocytes	Mild to moderate	0.01%–0.1%	Stinging of the skin, local dryness, hypopigmentation	Clinical trial (phase III)	Strong	Cosio et al. [15]
	Isotretinoin	Oral	Reduces sebaceous gland size, reduces sebum production, and decreases cell proliferation	Moderate to severe/nodulocystic acne	0.5–1 mg/kg/d	Decreased BMD, increased liver enzymes, hypertriglyceridemia, visual disturbance	Clinical trial (phase III)	Strong	Pile and Sadiq [16]
	Alitretinoin	Topical, oral	RXR and RAR agonist, Regulates the differentiation and proliferation of keratinocytes	Investigational in acne	10–30 mg/d (oral)	Increased LDL and triglycerides, decreased monocytes, headache	Preclinical	Weak	Rusu et al. [2]
	Acitretin	Oral	Agonist of all isotypes of RXR and RAR, regulates differentiation and proliferation of keratinocytes.	Not first‐line in acne; more for psoriasis	25–50 mg/d	Paresthesia, alopecia, xeroderma, pruritus, cheilitis	Preclinical	Weak	Zito et al. [17]
	Adapalene	Topical	RARβ and RARγ agonist regulates the differentiation of keratinocytes and keratinization.	Mild to moderate acne	0.1%–0.3%	Xeroderma, burning sensation of skin, exfoliation of skin, stinging of skin, erythema	Clinical trial (phase III)	Strong	Rusu et al. [2]
	Bexarotene	Topical, oral	RXR agonist regulates the differentiation and proliferation of keratinocytes	Investigational in acne	Unknown	Peripheral edema, headache, insomnia, skin rash, hyperlipidemia, hypothyroidism	Preclinical	Weak	Lowe and Plosker [18]
	Tazarotene	Topical	Modulates the differentiation and proliferation of epithelial tissue	Moderate to severe acne	0.05%–0.1%	Skin irritation, xeroderma, pruritus	Clinical trial (phase III)	Strong	Cosio et al. [15]
	Trifarotene	Topical	RARγ agonist regulates the differentiation of keratinocytes	Mild to moderate facial and truncal acne	0.005%	Sunburn, application‐site irritation	Clinical trial (phase III)	Strong	Rusu et al. [2]
		Antibacterials							
Lincosamides	Clindamycin	Topical, oral	Interacts with the 23S rRNA of the large 50S bacterial ribosomal subunit and inhibits the formation of peptide bonds and peptidyl tRNA movement in the ribosome.	Mild to moderate inflammatory acne	1%	Erythema, local dryness of skin, diarrhea, *Clostridioides difficile* infection	Clinical trial (phase III)	Strong	Armillei et al. [19]
Macrolides	Erythromycin	Topical, oral	Attaches to the 50S ribosomal subunit at the 23S rRNA site and inhibits the elongation of the growing peptide, leading to the dissociation of peptidyl tRNA molecules. Inhibits the release of synthesized peptides	Mild inflammatory acne	2%	Erythema, desquamation, local dryness of skin, QT_c_ prolongation, seizure, anorexia,	Clinical trial (phase III)	Strong	Armillei et al. [19, 20]
	Azithromycin	Oral	As erythromycin	Moderate acne	500 mg three times a week	QT_c_ prolongation, clostridioides difficile infection, ototoxicity	Clinical trial (phase III)	Strong	Armillei et al. [19, 20]
Tetracyclines	Doxycycline	Oral	Inhibits protein translation by attaching to the 16S rRNA of the 30S bacterial ribosomal subunit and inhibiting the entry of tRNAs into the A‐site	Moderate to severe acne	50–100 mg/d	Bone growth suppression, esophageal injury, photosensitivity, skin hyperpigmentation	Clinical trial (phase III)	Strong	Armillei et al. [19]
	Minocycline	Topical, oral	As doxycycline	Moderate to severe acne	50–100 mg/d (oral) 4% (topical)	Bone growth suppression, tinnitus, vertigo, intracranial hypertension, hepatotoxicity, photosensitivity, skin hyperpigmentation	Clinical trial (phase III)	Strong	Armillei et al. [19]
Sulfonamides	Sulfur and sodium sulfacetamide	Topical	Sulfur breaks disulfide bonds in skin cells. Sulfacetamide inhibits bacterial growth by inhibiting folic acid and DNA synthesis through competitive antagonism of PABA.	Mild acne	Sulfur: 2%–10%, Sulfacetamide: 10%	Erythema, Stevens‐Johnson syndrome, toxic epidermal necrolysis	Clinical trial (phase III)	Strong	Kim and Kim [1]
—	Benzoyl peroxide	Topical	Produces free radicals that can destroy the cell walls of *C. acnes* bacteria	Mild to moderate acne	2.5%–5%	Contact dermatitis, erythema, desquamation	Clinical trial (phase III)	Strong	Kim and Kim [1]
		Antimicrobials							
Para‐aminobenzoic acid antagonists	Dapsone	Topical	Inhibits the synthesis of dihydrofolic acid by attaching to the active site of dihydropteroate synthetase	Moderate inflammatory acne (esp. adult female)	5%	Sinusitis, facial edema, erythema	Clinical trial (phase III)	Strong	Kurien et al. [21]
—	Azelaic acid	Topical	Inhibits tyrosinase, mitochondrial respiratory chain enzymes, and DNA synthesis. Acts as a scavenger of free radicals, inhibits mitochondrial oxidoreductases, and 5α‐reductase	Mild to moderate acne	15%–20%	Burning sensation, stinging, and tingling of the skin	Clinical trial (phase III)	Strong	Sauer et al. [22]
		Keratolytic agent							
—	Salicylic Acid	Topical	Standardizes the microbial populations linked to the skin	Mild acne (esp. comedonal)	0.5%–2%	Localized irritation, desquamation, and exfoliation of skin	Clinical trial (phase III)	Strong	Bilal et al. [23]
		Novel treatments Hormonal regulators							
Androgen receptor inhibitors	Clascoterone	Topical	Androgen receptor antagonist	Moderate acne ( ≥ 12 years, both sexes)	1%	Adrenal suppression, xeroderma, and exfoliation of skin	Clinical trial (phase III)	Strong	Manjaly et al. [24]
		Antibacterials							
Tetracyclines	Sarecycline	Oral	As doxycycline	Moderate to severe acne (age ≥ 9)	1.5 mg/kg/d	Nausea, vulvovaginal infection	Clinical trial (phase III)	Strong	Armillei et al. [19]
		Miscellaneous							
Cannabinoids	Cannabidiol	Topical	CB2 receptors induce anti‐inflammatory properties by regulating the AMPK‐SREBP‐1 pathway, which reduces lipid production	Mild to moderate acne (investigational)	1%–3%	Skin rash, weight loss, diarrhea, vomiting, anemia, fever, viral infection, drowsiness	Clinical trial (Phase II)	Strong	(Lee et al. [25])
5‐Lipoxygenase inhibitors	Zileuton	Oral	Inhibits 5‐lipoxygenase and formation of LTB (4)	Moderate to severe acne (off‐label)	600 mg QID	Headache, dyspepsia, myalgia, abdominal pain	Clinical trial (phase III)	Strong	Zouboulis [26]
LTA4H inhibitors	Acebilustat	Oral	Inhibits LTA4H	Moderate acne	100 mg/d	Headache, cough, oropharyngeal pain, sputum production	Clinical trial (Phase II)	Strong	Gollnick et al. [27]
—	Lupeol	Topical	By modifying the IGF‐1R/PI3K/Akt/SREBP‐1 signaling pathway in SEB‐1 sebocytes, it represses lipogenesis. Reducing the NF‐κB pathway in SEB‐1 sebocytes and HaCaT keratinocytes reduces inflammation.	Moderate acne	0.2%–1%	Skin irritation	Clinical trial (phase III)	Strong	Kwon et al. [28]
—	Olumacostat glasaretil	Topical	Inhibits acetyl‐Coenzyme A carboxylase, decreases fatty acyl chains in sebum lipids, and reduces sebaceous gland size	Moderate acne	7.5%	Skin irritation	Clinical trial (phase III)	Strong	Melnik [29]
Azole	Talarozole	Topical	Inhibits CYP26, a key enzyme in the metabolism of RA	Mild to moderate acne	0.35%–0.7%	Skin irritation	Preclinical	Weak	Valente Duarte de Sousa [30]
—	Epigallocatechin3‐gallate	Topical	Reduces sebaceous gland size, sebocyte count, and comedo size, suppresses cell proliferation and lipid production in SZ95 sebocytes by blocking IGF‐1, decreases 5a‐reductase‐1 activity, reduces lipogenesis in human SEB‐1 sebocytes, reduces inflammation by inhibiting the NF‐kB and AP‐1 pathways, and promotes cytotoxicity in SEB‐1 sebocytes via apoptosis.	Mild to moderate acne (preclinical and pilot trials)	1%–2%	Skin irritation, liver and kidney failure	Clinical trial (phase III)	Strong	Im et al. [31, 32, 33]
—	Botulinum toxin type A	Intradermal injection	Suppresses sebum production in sebaceous glands by preventing the release of acetylcholine in sebocytes	Moderate to severe acne (off‐label)	2–5 units per site	Pain, bruising, and swelling at the injection site, headache	Clinical trial (phase III)	Strong	Birkett et al. [34]
Nanoparticles	Zinc oxide	Topical/in‐fiber	Antimicrobial, anti‐inflammatory via ROS and membrane disruption	Mild to moderate acne	≤ 1% in polymeric fibers	Cytotoxicity, genotoxicity, and inflammation	Clinical trial (phase III)	Strong	[35, 36, 37]

Abbreviations: AMPK, adenosine monophosphate‐activated protein kinase; BMD, bone marrow density; *C. acnes*, *Cutibacterium acnes*; CB2, cannabinoid receptor 2; DNA, deoxyribonucleic acid; IGF‐1, insulin‐like growth factor‐1; LTA4H, leukotriene A4 hydrolase; LTB (4), leukotriene B (4); NF‐κB, nuclear factor‐κB; PI3K, Phosphatidylinositide 3 kinase; RA, retinoic acid; RAR, retinoic acid receptor; rRNA, ribosomal ribonucleic acid; RXR, retinoid X receptors; SREBP‐1, sterol regulatory element‐binding protein‐1; SEB‐1, sebocyte‐1; tRNA, transfer ribonucleic acid.

### 2.2 Retinoids

**Figure 1:**
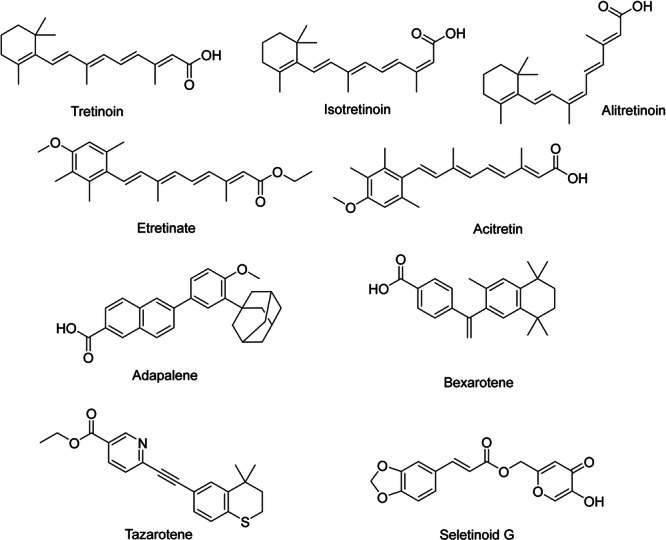
Chemical structures and brief pharmacology of synthetic retinoids; Tretinoin: First‐generation retinoid; all‐*trans*‐retinoic acid. Binds RAR‐α, ‐β, ‐γ. Induces epidermal hyperplasia, modulates keratinocyte differentiation; FDA‐approved for acne vulgaris and photoaging; Isotretinoin: 13‐*cis*‐retinoic acid isomer. Potent sebum suppression via sebocyte apoptosis regulates follicular keratinization. Gold standard for severe nodulocystic acne; Alitretinoin: 9‐*cis*‐retinoic acid. Pan‐RAR + RXR agonist. Unique indication: topical/oral therapy for chronic hand eczema and AIDS‐related Kaposi sarcoma; Etretinate: Aromatic retinoid (second‐generation). Prolonged tissue retention due to lipophilicity. Replaced by acitretin due to teratogenicity and long half‐life; Acitretin: Active metabolite of etretinate. Inhibits aberrant keratinization in psoriasis; used in pustular and erythrodermic variants. Teratogenic (3‐year contraceptive requirement post‐therapy); Adapalene: Third‐generation polyaromatic retinoid. Selective RAR‐β/γ agonist. Photostable, less irritant; preferred in acne for anti‐comedogenic and anti‐inflammatory effects; Bexarotene: Rexinoid (selective RXR agonist). Targets CTCL (cutaneous T‐cell lymphoma) via apoptosis induction in malignant T‐cells; also used in refractory psoriasis; Tazarotene: Prodrug converted to tazarotenic acid. High‐affinity RAR‐β/γ agonist. Normalizes keratinocyte differentiation; indicated for plaque psoriasis and acne; Seletinoid G: Novel fourth‐generation selective RAR‐γ agonist (investigational). Minimizes classical retinoid irritation while retaining anti‐proliferative efficacy in psoriasis models.

Retinoids regulate the keratinization, differentiation, maturation, and proliferation of epidermis [15]. In addition, they prevent hyperproliferation in the epithelium of the sebaceous glands' excretory ducts and reduce the formation of debris [38]. The retinoid group includes vitamin A (retinol), its natural derivatives (retinaldehyde, retinyl ethers), and numerous synthetic derivatives. Retinoids are typically classified based on generations. Vitamin A and certain synthetic derivatives, such as Tretinoin (ATRA; all‐trans retinoic acid) (Figure 1), Isotretinoin (13cRA; 13‐cis‐retinoic acid) (Figure 1), and Alitretinoin (9cRA; 9‐cis‐retinoic acid) (Figure 1), are part of the first generation of retinoids. The second generation includes Etretinate (Figure 1) and Acitretin (Figure 1), which feature an aromatic cyclic moiety in their chemical makeup. The third generation has polyaromatic compounds like Adapalene (ADP) (Figure 1), Bexarotene (Figure 1), and Tazarotene (Figure 1) [2]. Unlike the first generation, they act selectively [15]. Trifarotene was recently approved as the fourth generation of retinoids [2]. Seletinoid G (Figure 1) is another fourth‐generation retinoid. It is a pyranone derivative [39]. By moving from the first to the fourth generation, toxicity decreases, stability and efficacy increase [1]. First‐generation retinoids are commonly utilized for acne treatment and in anti‐aging cosmetic products. Second‐generation retinoids are utilized as systemic medications for treating psoriasis and dermatoses. Third‐generation retinoids are used in dermatology and oncology [38].

**Figure 2:**
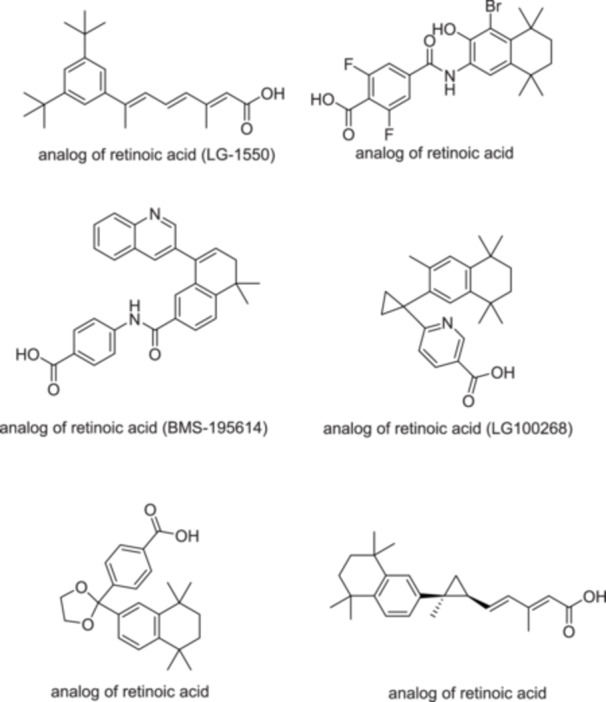
Chemical structures and brief pharmacology of selected synthetic retinoic acid analogs (investigational or discontinued); LG‐1550: Aromatic retinoid analog with halogen substitutions (Br, F). Selective RAR agonist; explored for reduced hypervitaminosis A toxicity while maintaining anti‐proliferative effects in skin disorders; BMS‐195614: Benzoimidazole‐based retinoid. High‐affinity RAR‐α antagonist; investigated for anti‐cancer potential via blockade of retinoid signaling in tumor cells; LG100268: Alkyne‐linked rexinoid. Potent, selective RXR agonist; studied in metabolic syndromes and cancer for RXR‐mediated apoptosis without classic RAR side effects; Unnamed analog (top‐right): Difluorinated aromatic retinoid. Designed for enhanced photostability and receptor selectivity; preclinical data suggest anti‐inflammatory activity in acne models; Unnamed analog (bottom‐left): Tricyclic polyene system. Rigidified retinoic acid mimic; aimed at improving RAR‐β/γ specificity for psoriasis with lower irritation; Unnamed analog (bottom‐right): Conformationally restricted alicyclic retinoid. Locks *trans*‐configuration; under evaluation for sustained keratinocyte normalization in ichthyosis.

The group of retinoid nuclear receptors includes retinoic acid receptors (RARs), which naturally bind to retinoic acid, and retinoid X receptors (RXRs), which naturally bind to 9cRA [2]. RARs have three types: RARα, RARβ, and RARγ. RARα has a Ser232 residue as a hydrogen bond donor in the LBP. In comparison, RARβ and RARγ have Ala225 and Ala234 as lipophilic residues in the LBP, respectively. RARγ has Met272 as a slightly polar residue in the LBP, so a weak hydrogen bond can form between this particular residue and retinoids that have a hydrogen‐bond donor close to the hydrophobic region of LBP [39]. Two hydrophobic regions, two aromatic structures, and negative ionic characteristics are essential pharmacophores for binding to RARγ. RARγ is predominantly expressed in the skin, which is implicated in conditions such as psoriasis and acne [40]. Each type of RAR has several isoforms, like RARα1 and RARα2, RARβ1 to RARβ4, and RARγ1 and RARγ2. Furthermore, there are three RXR isotypes: RXRα, RXRβ, and RXRγ. These isotypes have identical residues in the LBPs. Each RXR isotype consists of two isoforms: RXRα1 and RXRα2, RXRβ1 and RXRβ2, and RXRγ1 and RXRγ2 [39]. RARs are associated with keratinocyte proliferation and differentiation, while RXRs are associated with apoptosis [2]. Specific cytoplasmic retinol‐binding proteins (CRBPs) and cellular retinoic acid‐binding proteins (CRABPs) regulate intracellular bioavailability and have a specific affinity for retinol and RA, respectively. The interactions between RA and its nuclear receptors are regulated by CRABPs [15]. Natural retinoids have three important parts in their structure: (1) a *p*‐ionone ring (a lipophilic moiety) [2], retinoids containing bulky groups such as *p*‐tolyl or biphenyl in their hydrophobic region interfere with the correct position within the ligand‐binding domain. The result of this interference is the stabilization of the receptor‐co‐repressor complex and suppression of gene transcription. Retinoids possessing these characteristics are referred to as inverse agonists or negative antagonists as they develop a repressive complex and suppress the receptor's transcriptional activity [39]. (2) an isoprene chain sensitive to isomerization. In addition, double bonds have an important role [2]. (3) A polar moiety that is susceptible to oxidation [2]. To effectively attach to the LBPs of the RAR or the RXR, the polar moiety of the retinoid should have the capability to establish beneficial interactions with the amino acid residues located at the LBP [39]. First and second‐generation retinoids are flexible molecules and have a non‐selective effect. The rigidity that was developed in the third‐generation retinoids, by adding aromatic rings, created more selective molecules [2]. RAR isotype‐specific retinoids (synthetic retinoids) have less toxicity than natural retinoids like 9cRA and ATRA [39]. Synthetic retinoids have a benzene ring in place of the cyclohexane in the natural vitamin A structure [15]. Arotinoids are synthetic retinoids that consist of at least one aromatic ring. Numerous arotinoids exhibit a similar structural pattern, with 1,1,4,4‐tetramethyl‐1,2,3,4‐tetrahydronaphthalene as the hydrophobic component and a carboxylate‐bearing aromatic ring as the polar end. The short linker unit, usually composed of 1–3 atoms, connects these two parts. This linker regulates selectivity between RXRs, RARs, and their various isotypes [39]. ADP is a naphthoic acid. It attaches more to RARγ and RARβ [2]. It does not interact with CRABPs [38]. ADP has four bonds that can rotate, and it doesn't have any stereoisomers. The adamantane nucleus is a very important part of the ADP structure, facilitating the attachment of ADP to RARγ and RARβ. Changing the 4‐methoxyphenyl of ADP to a 4‐hydroxyphenyl creates analogs that have antimicrobial effects against Methicillin‐resistant *Staphylococcus aureus* (MRSA) and *Enterococcus faecalis*. The 4‐methoxyphenyl group has antiproliferative properties [2]. Isotretinoin, an orally administered RA derivative, manages severe refractory nodulocystic acne. Its primary mechanism of action involves the decrease of sebaceous gland size and the inhibition of sebum production, thereby modifying the lipid composition of the skin's surface. Substituting tretinoin's functional groups with stronger pharmacophores makes it possible to create retinoids that are more durable against isomerization and metabolism, resulting in enhanced overall efficacy. For example, conjugated tetraene and trimethylcyclohexenyl rings can be substituted with more durable structural units like biaryl, stilbene, or tolan [39]. Here, we have some analogs of retinoic acid (Figure 2) [41].

### 2.3 Lincosamides

**Figure 3:**
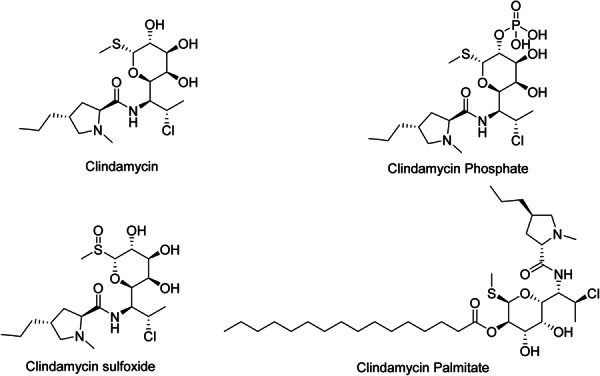
Chemical structures and brief pharmacology of Clindamycin and its analogs used in acne treatment; Clindamycin: Lincosamide antibiotic; 7(S)‐chloro‐7‐deoxylincomycin. Inhibits bacterial protein synthesis by binding 50S ribosomal subunit. Topical/oral gold standard for acne vulgaris (anti‐*Cutibacterium acnes*); reduces inflammation via inhibition of pro‐inflammatory cytokines; Clindamycin phosphate: Phosphorylated prodrug. Inactive until dephosphorylated by cutaneous phosphatases to active clindamycin. Enhanced aqueous solubility; preferred in topical gels/foams for acne and rosacea; Clindamycin sulfoxide: Oxidized metabolite at sulfur. Inactive; formed via hepatic metabolism. Minimal clinical use; studied as a potential less‐irritating derivative; Clindamycin palmitate: Ester prodrug with palmitic acid. Hydrolyzed by esterases to active clindamycin. Used in pediatric oral suspensions; not indicated in dermatology due to poor skin penetration.

Lincosamides consist of a thiomethyl amino‐octoside connected by an amide bond to a propyl‐substituted *N*‐methylpyrrolidylcarboxylic acid. Lincomycin is a natural substance and is utilized as the initial component for the production of Clindamycin. The replacement of the hydroxyl group with a chloride resulted in the creation of Clindamycin, which is more bioactive and lipophilic compared to lincomycin [42]. Clindamycin (Figure 3), a semi‐synthetic antibiotic belonging to the lincosamide family, is commonly utilized for the treatment of skin issues such as AV. Clindamycin has activity against *C. acnes*, which is a Gram‐positive, anaerobic bacterium and contributes to the pathogenesis of acne. Clindamycin interacts with the 23S rRNA of the large 50S bacterial ribosomal subunit and inhibits the formation of peptide bonds and peptidyl tRNA movement in the ribosome. It forms hydrogen bonds with 23S rRNA residues [19]. In addition, we have some analogs of Clindamycin (Figure 3).

### 2.4 Macrolides

**Figure 4:**
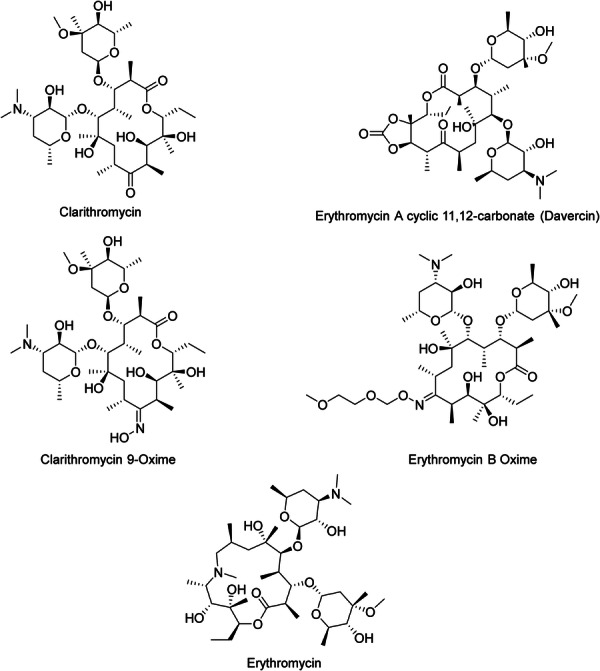
Chemical structures and brief pharmacology of Erythromycin and Clarithromycin, and selected analogs; Erythromycin: 14‐membered macrolide; binds 50S ribosomal subunit (domain V). Bacteriostatic against *Cutibacterium acnes* and *Staphylococcus* spp. Topical/oral use in acne; anti‐inflammatory via inhibition of neutrophil chemotaxis and cytokine release; Clarithromycin: 6‐*O*‐methyl erythromycin. Enhanced acid stability, improved oral bioavailability. Superior activity against *C. acnes*; used in acne and rosacea when erythromycin resistance emerges; Erythromycin Acyclic 11,12‐Carbonate (Davericin): semisynthetic derivative with carbonate bridge. Investigational; designed for reduced gastrointestinal motility side effects while retaining antimicrobial potency; Clarithromycin 9‐oxime: Oxime prodrug intermediate. Increased lipophilicity; precursor in the synthesis of clarithromycin—enhances tissue penetration in skin infections; Erythromycin B oxime: Modified at C‐9 ketone. Reduced bitterness and improved stability; explored as an intermediate for next‐generation macrolides with lower resistance induction.

Macrolides consist of a macrocyclic lactone with varying ring sizes, to which one or more deoxy‐sugar or amino sugar residues are linked. Erythromycin contains a 14‐membered lactone ring, and Azithromycin contains a 15‐membered one. The second‐generation derivatives of erythromycin, such as Azithromycin, incorporate all modifications at the C6 or C9 positions of the lactone ring. This alteration prevents the formation of the 9,12‐ and/or 6,9‐hemiketal forms, which decompose into spiroketal inactive derivatives, thus demonstrating resistance to acid‐catalyzed inactivation [43]. Macrolides attach to the 50S ribosomal subunit at the 23S rRNA site and inhibit the elongation of the growing peptide beyond a few residues, leading to the dissociation of peptidyl tRNA molecules [20]. Also, they inhibit the release of synthesized peptides [19]. Erythromycin, its analogs, and Azithromycin (Figure 4) are used for the treatment of acne [1].

### 2.5 Tetracyclines

**Figure 5:**
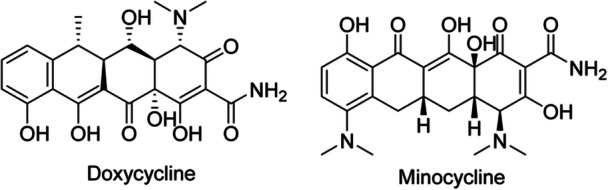
Chemical structures and brief pharmacology of Doxycycline and Minocycline in acne treatment; Doxycycline: Second‐generation tetracycline; 6α‐deoxy‐5‐hydroxy‐tetracycline. Inhibits *Cutibacterium acnes* protein synthesis (30S ribosome); potent anti‐inflammatory via ↓MMP‐9, ↓IL‐1β, ↓TNF‐α. First‐line oral therapy for moderate–severe acne and rosacea (sub‐antimicrobial 40 mg dose approved). Photosensitizer; once/twice‐daily dosing; Minocycline: Lipophilic second‐generation tetracycline; 7‐dimethylamino‐6‐demethyl‐6‐deoxytetracycline. Superior sebaceous gland penetration; highest anti‐*C. acnes* potency among tetracyclines. Preferred in nodulocystic acne; extended‐release 1 mg/kg minimizes vestibular side effects. Rare blue‐gray pigmentation (skin, teeth, sclera).

Tetracycline molecules are made up of a linear fused tetracyclic core (rings are labeled A, B, C, and D) with various functional groups attached. Important features necessary for having antibacterial activity in the tetracycline class of compounds are: preserve the linear fused tetracycle; the natural (α) stereochemistry at the 4a, 12a (A‐B ring junction), and 4 (the dimethylamino group); and preserve the keto‐enol system (11, 12 and 12a) near the phenolic d‐ring, in particular the aqueous moiety. Tetracyclines are potent chelating agents. The chelation sites are based on the β‐diketone system (positions 11 and 12), the enol groups (positions 1 and 3), and the carboxamide groups (position 2) within the A‐ring [44]. They obstruct protein translation by attaching to the 16S rRNA of the 30S bacterial ribosomal subunit. The binding of tetracycline to the 30S subunit disrupts the entry of incoming tRNAs into the A‐site, thereby significantly reducing protein translation in the *C. acnes* ribosome [19]. Doxycycline and Minocycline (Figure 5) are used for the treatment of acne [1].

### 2.6 Sulfur and Sodium Sulfacetamide

**Figure 6:**
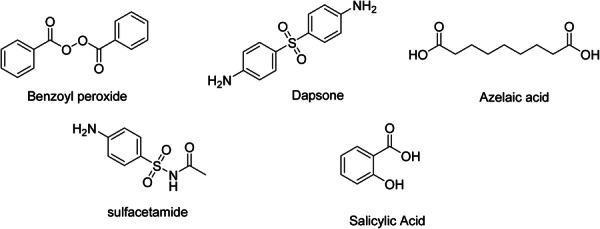
Chemical structures of benzoyl peroxide, dapsone, azelaic acid, salicylic acid, and sulfacetamide; Benzoyl peroxide: Lipophilic organic peroxide. Generates free radicals (•OH, •OOC₆H₅) that oxidize *C. acnes* proteins and cell membranes. Bactericidal, non‐resistance‐inducing; mild comedolytic and anti‐inflammatory. Gold standard 2.5%–10% gels/washes; may bleach fabrics; Dapsone: Sulfone antibiotic. Inhibits dihydropteroate synthase in *C. acnes*; anti‐inflammatory via ↓myeloperoxidase and ↓neutrophil ROS. Topical 5%–7.5% gel; minimal systemic absorption. Safe in G6PD deficiency (topical use); Azelaic acid: Dicarboxylic acid (C9). Competitive inhibition of tyrosinase ( ↓ melanin); normalizes keratinization; bacteriostatic versus *C. acnes*. 15%–20% cream; dual benefit in acne + post‐inflammatory hyperpigmentation. Minimal irritation; Salicylic acid: β‐Hydroxy acid. Lipophilic keratolytic; dissolves desmosomes in stratum corneum. Comedolytic at 0.5%–2%; anti‐inflammatory via COX inhibition. Preferred for oily skin and mild acne; Sulfacetamide: Sulfonamide antibiotic. Blocks folate synthesis in *C. acnes*. Often combined with sulfur (keratolytic synergy). 10% lotion; alternative in sulfa‐tolerant patients with inflammatory acne.

An unbound aromatic NH2 group located in the para‐position to the sulfonamide group is crucial for the efficacy of sulfonamides. N1‐monosubstituted derivatives of sulfanilamide are active agents, and their level of activity enhances with the addition of heteroaromatic substituents. Furthermore, the sulfonamide group must be directly connected to the benzene ring [45]. Sulfur‐based treatments are effective against the majority of *Staphylococcus* bacteria responsible for AV that have multidrug resistance [46]. It exerts its keratolytic effect by breaking disulfide bonds in skin cells. Sodium sulfacetamide (Figure 6) has a bacteriostatic effect and interferes with DNA synthesis [1]. Since it is a structural analog of PABA, it replaces it in the path of folic acid synthesis and inhibits cell division and proliferation. Adding an electron‐withdrawing substitution can enhance the antibacterial effect of sulfacetamide [47].

### 2.7 Benzoyl Peroxide

BPO (Figure 6) has been found to be effective as an antibacterial and comedolytic compound. However, BPO has been shown to be detrimental to the epidermal barrier through a few different mechanisms. For example, BPO is a very strong oxidizing agent that produces reactive oxygen species (ROS), which may denature cellular components found in the stratum corneum [48]. Oxidative stress may also affect intercellular lipids, including ceramides and free fatty acids that are essential for maintaining barrier function and hydration [49, 50]. In addition, low‐level chronic or high‐concentration dosing of BPO can contribute to increased transepidermal water loss (TEWL), subclinical inflammation, and erythema [51]. The broad‐spectrum antimicrobial effects of BPO may also disrupt microbial diversity on the skin surface, which can compromise the ecological niche of commensals [52]. The loss of diversity may be even more pronounced in sensitive patients, demonstrating contributions to skin homeostasis and therefore barrier fragility. While BPO remains a necessary, if not essential, agent in acne care, the formulation and frequency of dosing must be considered to optimize clinical efficacy with patient tolerability.

### 2.8 Dapsone

Dapsone is a competitive antagonist of PABA, and has para‐aminobenzene groups on the benzene rings that are essential for mimicking PABA. The sulfone (SO2) bridge between the two aminobenzene rings is also essential for the activity of dapsone [53]. Topical dapsone provides an effective alternative for treating acne. Dapsone (Figure 6) demonstrates superior efficacy in inflammatory acne compared to comedonal acne. It shows notable effectiveness in female patients and individuals aged 18 years and older [54]. Dapsone has a sulfone group in its structure. It has antimicrobial, anti‐inflammatory, and immunomodulatory properties [1].

### 2.9 Azelaic Acid

Azelaic acid (AZA) (Figure 6) is a naturally occurring saturated dicarboxylic acid that exhibits multiple therapeutic effects in acne treatment, many of which are closely linked to its acidic functional groups. These two carboxylic acid moieties allow AZA to interact with cellular components and enzymes at the skin surface and within follicles. Its acidic nature facilitates penetration into the pilosebaceous unit, where it can normalize abnormal keratinization, thereby preventing comedone formation. Moreover, the acidic pKa of AZA (around 4.5) contributes to the lowering of skin pH, creating a less hospitable environment for the proliferation of *Cutibacterium acnes*, and the topical AZA formulation typically has a pH range of approximately 3.8–4.0 [55]. Additionally, AZA selectively inhibits mitochondrial oxidoreductase and 5α‐reductase enzymes, partially due to the reactivity of its carboxylic acid groups, which disrupt oxidative processes within hyperproliferative keratinocytes and sebocytes. This results in anti‐inflammatory, antimicrobial, and anti‐keratinizing effects, making AZA effective for mild to moderate acne, especially in patients with post‐inflammatory hyperpigmentation or sensitive skin [56, 57, 58].

### 2.10 Keratolytic Agent

Keratolytic agents have been extensively utilized in the treatment of acne for several years. They demonstrate a swift enhancement in the management of acne vulgaris when compared to other treatment options [59].

### 2.11 Salicylic Acid

Salicylic Acid diminishes sebocyte lipogenesis by inhibiting the adenosine monophosphate‐activated protein kinase (AMPK)/sterol response element‐binding protein‐1 (SREBP‐1) pathway. It alleviates inflammation by suppressing the NF‐κB pathway in these cells. Additionally, salicylic acid reduced the viability of SEB‐1 sebocytes by triggering apoptosis via the death receptor pathway [60]. The presence of COOH and ortho‐OH groups is essential for the activity of the molecule and for maintaining the pKa of 2.97, and salicylic acid works best in a pH range of 3.0–4.0. The hydroxyl of the carboxyl group should not have any substituents [61]. Salicylic acid (Figure 6) has comedolytic, keratolytic, anti‐inflammatory, fungistatic, and bacteriostatic effects [1]. It is used in the non‐inflammatory lesions [62] and standardizes the microbial populations linked to the skin (Bilal et al. [23]).

## 3 Review of Novel Treatment Options by Their Chemical Structures

Conventional acne therapies, including antibiotics, retinoids, and hormonal agents, have shown clinical effectiveness, but many rely on strong‐acting ingredients that have unintentional side effects such as teratogenicity and other hormonal‐related issues, and could potentially lead to bacterial resistance or skin irritation. Innovative therapy agents have resulted in several developments that can help further minimize these issues. For example, compounds such as clascoterone have a dual‐action as a local androgen receptor antagonist and have no systemic hormonal effects, making them a safer option than oral antiandrogens [63]. Sarecycline is another innovative agent, which ultimately is a narrow‐spectrum tetracycline derivative, that has maintained the antibacterial properties of tetracycline while maximizing the avoidance of disruption to the gut and skin microbiomes, which can ultimately minimize resistance [64].

Novel agents such as Cannabidiol (CBD) and epigallocatechin‐3‐gallate (EGCG), which have multiple possible modes of action, and include aspects of anti‐inflammatory actions and sebosuppressive effects, may enhance tolerability and may benefit patients with sensitive skin or individuals wanting to include a botanical intervention in their treatment plan [33, 65]. Moreover, nanoparticle‐based formulations (e.g., ZnO‐loaded fibers) potentially enhance skin delivery, drug release, tolerability, irritation, and localized action (Rihova et al. [37]). These are examples of a shift towards precision dermatology. It will be highlighted that these agents and techniques are on‐target, microbiome‐friendly, and minimally invasive [66, 67]. As such, novel treatments not only expand the therapeutic toolbox but also provide safer and more personalized options for acne patients, particularly those who are unresponsive or intolerant to conventional drugs.

### 3.1 Clascoterone

**Figure 7:**
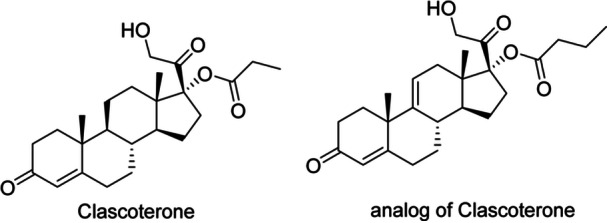
Chemical structures and brief pharmacology of clascoterone and its analog in acne treatment; Clascoterone: Cortexolone‐17α‐propionate; non‐systemic topical androgen receptor (AR) inhibitor. Competitive antagonist at dermal AR in sebocytes/pilosebaceous unit; ↓DHT‐driven sebum production and inflammation. FDA‐approved 1% cream for acne vulgaris (ages ≥ 12); minimal HPA‐axis suppression; Analog of Clascoterone: 11β‐hydroxy cortexolone derivative. Preclinical; enhanced AR affinity and sebostatic potency versus parent. Investigational for androgenetic alopecia and seborrheic dermatitis; improved follicular targeting.

Clascoterone (Figure 7) is a new topical antiandrogen drug licensed for the treatment of acne. Clascoterone has a similar structure to steroids and is a competitive antagonist of androgen receptors [68]. Conventional oral antiandrogen therapies for acne, such as a combination of oral contraceptives and spironolactone, have systemic hormonal effects that often prevent their use in male patients while limiting their use in some female patients. Conversely, Clascoterone is a first‐in‐class antiandrogen, and it is both safe and efficacious in female and male patients over the age of 12. Clascoterone is generally well‐tolerated, except for occasional localized skin irritation. Nevertheless, some teenagers in the Phase II clinical trial showed biochemical evidence of Hypothalamic‐pituitary‐adrenal (HPA) suppression, which resolved once therapy was discontinued [24]. Additionally, 9,11‐Dehydrocortexolone 17alpha‐butyrate is a Clascoterone analog (Figure 7).

### 3.2 Trifarotene

**Figure 8:**
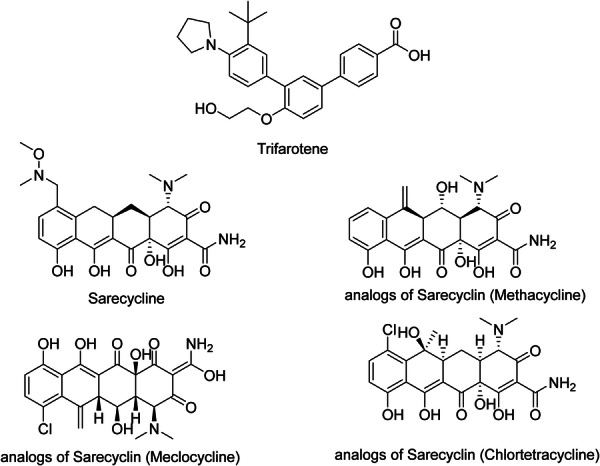
Chemical structures and brief pharmacology of trifarotene, sarecycline, and selected sarecycline analogs in acne treatment; Trifarotene: fourth‐generation retinoid; rigid polyaromatic scaffold with terminal carboxylic acid. Selective RAR‐γ agonist (100‐fold > RAR‐β/α). Potent normalization of keratinocyte differentiation and ↓inflammation in the pilosebaceous unit. FDA‐approved 0.005% cream for facial/trunk acne; superior comedolytic efficacy versus tretinoin with reduced irritation; Sarecycline: narrow‐spectrum tetracycline; C7‐piperidinyl substitution. High *C. acnes* potency, minimal GI dysbiosis ( ↓ *Enterococcus*, *Escherichia*). Anti‐inflammatory via ↓MMP‐13, ↓IL‐6. FDA‐approved 1.5 mg/kg oral for moderate–severe non‐nodular acne; weight‐based dosing; Methacycline: C6‐methylene derivative. Broader‐spectrum; precursor in tetracycline synthesis. Higher photosensitivity; limited modern use due to resistance; Meclocycline: C7‐chloro‐6‐methylene. Enhanced lipophilicity; investigational topical. Poor stability; discontinued; Chlortetracycline: first‐generation; C7‐chloro. Broad Gram‐positive coverage; high resistance rates. Historical use; replaced by doxycycline/minocycline.

As mentioned previously, Trifarotene (Figure 8) is a unique fourth‐generation locally administered retinoid. It is approved for the first time in treatment regimens for both facial and truncal acne [69]. Trifarotene is a topical retinoid that targets RARγ. Trifarotene's high selectivity for RAR‐γ in skin results in potency even at low concentrations. Trifarotene specifically targets RAR‐γ receptors 20 times greater than that of RAR‐α and RAR‐β receptors [2]. After binding to the RAR‐γ, it becomes a dimer. Trifarotene does not target RXRs [15]. It is used to treat acne [40] and has anti‐inflammatory and comedolytic properties [15]. Trifarotene, like other topical retinoids, works by boosting keratinocyte differentiation while lowering proliferation and hyperkeratinization. Retinoids have also been demonstrated to block inflammatory processes by targeting leukocyte migration, toll‐like receptors (TLRs), and Activator Protein (AP)−1 [70].

### 3.3 Sarecycline

Sarecycline (Figure 8) is an oral, once‐daily, tetracycline‐class medication licensed in the United States for the inflammation‐related areas of non‐nodular moderate to severe AV in individuals aged 9 years or older [71]. Sarecycline has anti‐inflammatory effects. It has a powerful effect against Gram‐positive bacteria, including numerous strains of *C. acnes*, while having little activity against enteric aerobic Gram‐negative bacteria [64]. This gives it a more selective antibacterial spectrum and reduces the possibility of unwanted off‐target antibacterial effects, giving it a more viable therapy option than others in the tetracycline class. There is not much resistance against it compared to other tetracyclines. Also, it is effective against tetracycline‐resistant *S. aureus* as well as erythromycin‐ and clindamycin‐resistant *C. acnes* strains [72]. In Figure 8, some analogs of Sarecyclin are demonstrated.

### 3.4 Cannabidiol

**Figure 9:**
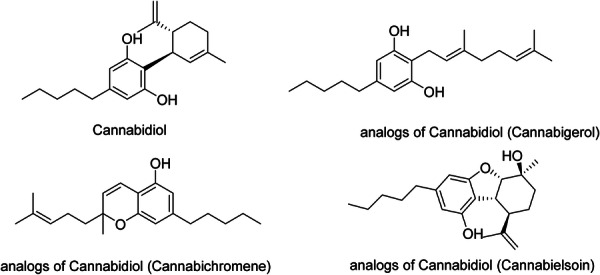
Chemical structures and brief pharmacology of cannabidiol (CBD) and selected analogs in acne treatment; Cannabidiol (CBD): non‐psychoactive phytocannabinoid; resorcinol with pentyl side chain. CB2 > CB1 partial agonist, GPR55 antagonist, TRPV1 agonist. ↓Sebocyte lipogenesis, ↓TLR4/NF‐κB‐mediated inflammation, and ↑elastin/collagen III. Topical 1%–3% investigational for acne, atopic dermatitis, psoriasis; antioxidant, anti‐pruritic; Cannabigerol (CBG): biosynthetic precursor; monoterpenoid phenol. Potent α2‐adrenergic agonist, 5‐HT1A antagonist. Anti‐inflammatory via ↓PGE2; early data suggest superior anti‐acne sebum suppression versus CBD; Cannabichromene (CBC): propyl‐side‐chain variant. TRPA1/TRPV3 agonist, with minimal CB receptor affinity. Enhances wound healing via ↑keratinocyte migration; potential synergy with CBD in eczema; Cannabielsoin (CBE): metabolite with a fused oxepane ring. Low CNS penetration; preclinical anti‐fibrotic via ↓TGF‐β in dermal fibroblasts. Explored for scleroderma and hypertrophic scars.

CBD (Figure 9) is a nonpsychoactive phytocannabinoid found in the *Cannabis sativa* (hemp) plant. CBD plays a role in the treatment of a variety of inflammatory illnesses, including cancer, dementia, immunological disorders, and dermatological conditions. CBD and cannabis seeds were reported to lower inflammation and expression of inflammatory cytokines, like tumor necrosis factor‐alpha (TNF‐α) and interleukin (IL)−1β, in acne‐like situations. Treatment with these cannabis extracts was likewise shown to be safe and well‐tolerated [73]. CBD had a concentration‐dependent effect on cell viability, significantly reducing SEB‐1 sebocytes' viability; moreover, it triggered apoptosis and a considerable increase in the apoptotic region at higher doses. CBD significantly lowered pro‐inflammatory cytokines like C‐X‐C Motif Chemokine Ligand 8 (CXCL8) and IL‐1α. It also suppressed lipid production by regulating the AMPK‐SREBP‐1 pathway and substantially decreased the hyperkeratinization‐associated protein keratin 16. CBD also promotes the production of elastin, collagen 1, and collagen 3 [25]. CBD has some derivatives and analogs (Figure 9).

### 3.5 Zileuton

**Figure 10:**
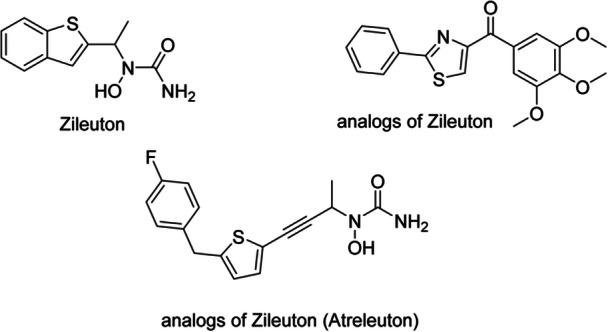
Chemical structures and brief pharmacology of zileuton and selected analogs in acne treatment; Zileuton: *N*‐hydroxyurea 5‐lipoxygenase (5‐LOX) inhibitor; benzothiophene core. ↓LTB4, ↓LTC4/D4/E4 synthesis in the arachidonic acid cascade. Reduces neutrophil chemotaxis and inflammation. Oral 600 mg BID; off‐label for acne fulminans, hidradenitis suppurativa; hepatotoxicity monitoring required; Analog of Zileuton (unsubstituted benzothiophene derivative): Simplified scaffold with methoxy terminus. Preclinical; retains 5‐LOX inhibition with improved oral bioavailability. Investigational for chronic urticaria and neutrophilic dermatoses; Analog of Zileuton (Atreleuton, ABT‐761): fluorinated phenyl variant. Enhanced potency (IC₅₀ ~0.05 μM) and longer half‐life versus zileuton. Phase II/III for asthma; dermatologic potential in leukotriene‐driven disorders (e.g., eosinophilic cellulitis).

Tissue inflammation is a crucial part of the acne process. Leukotriene B(4) (LTB(4)) may cause the inflammation of tissues. The enzyme 5‐lipoxygenase controls the synthesis of LTB(4). Zileuton (Figure 10) inhibits the activity of 5‐lipoxygenase; hence, experimental and clinical investigations have been done to determine its method of action, effectiveness, and safety in treating AV [26].

### 3.6 Acebilustat

**Figure 11:**
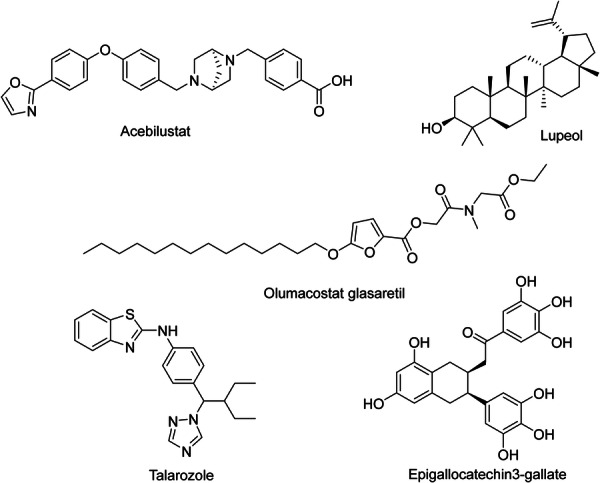
Chemical structures and brief pharmacology of emerging topical/oral agents targeting sebaceous gland function in acne treatment; Acebilustat: benzoxazolyl‐phenylsulfonamide. LTB4 synthesis inhibitor (BLT1/2 antagonist). ↓Neutrophil recruitment, ↓IL‐1β. Phase II oral for moderate–severe acne; reduces inflammatory lesions without systemic immunosuppression; Lupeol: pentacyclic triterpenoid (betulin skeleton). Inhibits 5α‐reductase type 1 in sebocytes; ↓DHT‐driven lipogenesis. Anti‐inflammatory via NF‐κB/STAT3. Plant‐derived; preclinical topical for acne and seborrhea; Olumacostat glasaretil: acetyl‐CoA carboxylase (ACC) prodrug inhibitor. Blocks de novo fatty acid synthesis in sebocytes. ↓Sebum triglycerides. Phase II topical gel; development halted due to efficacy plateau; Talarozole: azole‐based CYP26 inhibitor. ↑Endogenous all‐*trans*‐retinoic acid in skin. Normalizes keratinization, ↓sebum. Oral 1–2 mg; phase II for acne and ichthyosis; mild retinoid‐like side effects; Epigallocatechin‐3‐gallate (EGCG): green tea catechin gallate ester. ↓SREBP‐1, ↓IGF‐1 signaling in sebocytes. Potent antioxidant, anti‐comedogenic. Topical 1%–3% unstable; encapsulated formulations in clinical trials for acne.

Acebilustat (Figure 11) is the only selective leukotriene A4 hydrolase (LTA4H) inhibitor presently in clinical trials for acne treatment. A phase 2 experiment evaluated the effectiveness of once‐daily oral Acebilustat therapy on lesion counts in 124 individuals with moderately severe face acne vulgaris. Acne patients receive either 100 mg Acebilustat or a placebo for 12 weeks in a 2:1 ratio. This trial has been finished but has not yet been reported [27].

### 3.7 Lupeol

After experimental examination, a pentacyclic triterpene called Lupeol (Figure 11) was found in the hexane extract of *Solanum melongena*. Lupeol addressed the majority of acne's primary pathogenic aspects while retaining desirable physicochemical properties. It repressed lipogenesis by modifying the IGF‐1R/phosphatidylinositide three kinase (PI3K)/Akt/SREBP‐1 signaling pathway in SEB‐1 sebocytes. It also lowered inflammation by reducing the NF‐κB pathway in SEB‐1 sebocytes and HaCaT keratinocytes. Lupeol had a minor influence on cell survival and may have modified epidermal dyskeratosis. Histopathological analysis of human acne tissues after 4 weeks of lupeol treatment revealed that lupeol significantly reduced the number of infiltrated cells and major pathogenic proteins examined in vitro, surrounding comedones or sebaceous glands, providing solid evidence for the suggested therapeutic mechanisms. These findings illustrate the therapeutic viability of using lupeol to treat acne [28].

### 3.8 Olumacostat Glasaretil

Olumacostat glasaretil (OG) (Figure 11), an acetyl coenzyme A carboxylase inhibitor, decreases saturated and monounsaturated fatty acyl chains in sebum lipids. Topical OG treatment reduces hamster ear sebaceous gland size and is effective in treating acne vulgaris. OG‐mediated sebum reduction may inhibit P. acnes development and biofilm formation, as well as comedogenesis and inflammation [29]. Also, in a clinical trial, OG was well tolerated and shown to be effective, indicating that further research is required [74].

### 3.9 Talarozole

Talarozole (Figure 11), also known as Rambazole or R115866, is a selective azole derivative that inhibits CYP26, a key enzyme in the metabolism of RA. RA has a role in comedo development by influencing keratinization inside the follicular epithelium. Rat studies have indicated that high plasma levels of RA are related to the suppression of keratinization. In a Phase I randomized experiment with 16 healthy persons, a gel formulation with 0.35% and 0.7% Talarozole was found to have RA benefits while causing reduced inflammation. Even after a single topical treatment, Talarozole has been shown to have retinoid‐like effects [30].

### 3.10 Epigallocatechin3‐Gallate

EGCG (Figure 11), a polyphenol found in green tea, has been shown in animal models to lower sebaceous gland size, sebocyte count, and comedo size, as all‐trans‐retinoic acid does. Furthermore, EGCG suppresses cell proliferation and lipid production in SZ95 sebocytes in vitro by blocking IGF‐1 [31]. EGCG can decrease 5a‐reductase‐1 activity, reducing DHT‐dependent sebum production [32]. Furthermore, EGCG shows antibacterial action against *P. acnes* and is therapeutically beneficial in treating acne with minimal adverse effects. In addition, EGCG can reduce lipogenesis in human SEB‐1 sebocytes by 55% in vitro, reduce inflammation by inhibiting the NF‐kB and AP‐1 pathways, and promote cytotoxicity in SEB‐1 sebocytes via apoptosis [33].

### 3.11 Botulinum Toxin Type A

**Figure 12:**
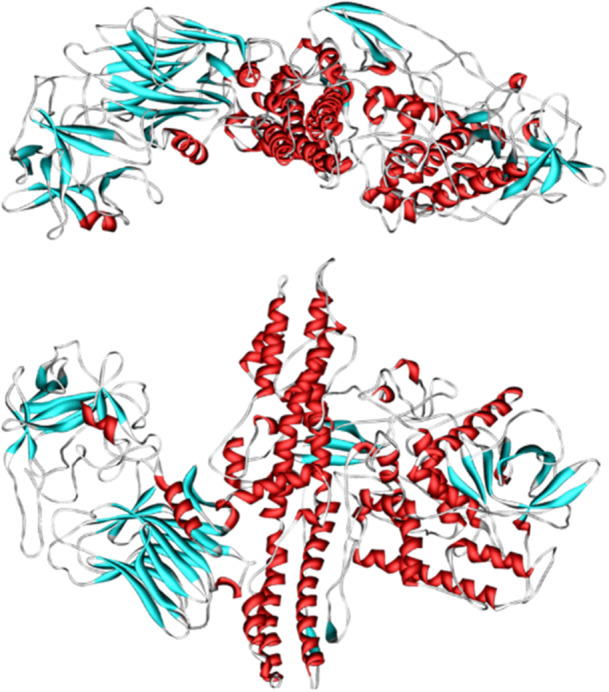
3D structure of botulinum neurotoxin.

Botulinum neurotoxin (Figure 12) is a protein dimer with a molecular weight of 150 kDa and the chemical formula C_6760_H_10447_N_1743_O_2010_S_32_, composed of two chains: light and heavy. The light chain accounts for around one‐third of the toxin's molecular weight and is linked to the heavy chain via a disulfide bond [75]. Botulinum toxin type A (BTX‐A) suppresses sebum production in sebaceous glands by preventing the release of acetylcholine in sebocytes and eliciting flaccid paralysis in the arrector pili smooth muscle of follicular units [34]. There is growing evidence that acetylcholine plays specialized functions in sebum production, implying that BTX‐A may inhibit sebum production by interrupting cholinergic transmission between sebaceous glands and autonomic nerve terminals. BTX‐A also suppresses various pathogenetic components of acne development, implying that it can be employed as a safe and effective therapy option for acne and other skin problems caused by excessive sebaceous gland activity [76].

### 3.12 Zinc Oxide Nanoparticles

Zinc oxide nanoparticles (ZnO NPs) have become an attractive adjunctive therapy for acne vulgaris in recent decades, largely due to their broad‐spectrum antimicrobial activity targeting *C. acnes* (previously *Propionibacterium acnes*) and anti‐inflammatory properties. ZnO NP efficacy is structure‐dependent, with small particle size (< 20 nm) and high surface area‐to‐volume ratios enhancing the formation of ROS like hydrogen peroxide (H_2_O_2_) and hydroxyl radicals (•OH) that rupture bacterial membranes and intracellular constituents [35]. To maximize bioavailability, ZnO NPs can be incorporated into biopolymeric fibers (e.g., polyvinyl alcohol) with centrifugal spinning technology, which can provide optimal skin contact, sustained NP release with minimal risk of aggregation, and significantly enhance antibacterial efficacy against *C. acnes* biofilms in addition to reducing sebum‐induced inflammation (Rihova et al. [37]). Most recently, clinical ex vivo studies have shown fiberglass infused with ZnO NPs reduced acne lesions by > 60% and TNF‐α by > 30% with little cytotoxicity to keratinocytes, further supporting the viability of these NPs as a biocompatible, antibiotic‐free treatment strategy [36, 77].

## 4 Effective Bioactive (Natural) Compounds in Acne Treatment

Nowadays, due to the side effects of certain drugs, consumers prefer to use natural ingredients for treating diseases. A broad spectrum of bioactive molecules in plant, animal, and mineral sources are known as natural compounds, which may have the potential to treat acne. Herbal or medicinal plants (extracts, powders, and essential oils) are considered preferable to treat acne owing to their bioactive compounds and likely negligible adverse effects [78]. According to early‐stage evidence, phenolic constituents reveal growth inhibitory activity against *P. acnes* through their anti‐inflammatory, antioxidant, and antimicrobial features, therefore displaying an anti‐acne action [79]. Previous research confirmed the antiacne effect of aloe vera [80] with an in vitro study, barberry [81, 82] by clinical experiments, safflower [83] with an in vitro survey, ginger and turmeric [84], bromelain and curcumin for treating acne scars [85] with clinical trials, via their phenolic compounds such as aloin, emodin, anthocyanins, flavonoids, epigallocatechin‐3‐gallate, curcumin, gingerols and shogaols [79]. Terpenoids and steroids are other beneficial compounds that are likely to have effects on acne treatment related to their antioxidant, anti‐inflammatory, and antibacterial properties [78, 86]. In a clinical study targeting the treatment of acne vulgaris, rosemary essential oil was efficient in decreasing acne lesions, and its terpenoid compounds, such as rosmarinic acid, carnosol, and carnosic acid, were involved in improving acne [87]. Additionally, another active compound in acne treatment belongs to alkaloids [86]. Numerous investigations have been conducted to determine the anti‐acne activity of alkaloids [79, 88, 89]. Based on early‐stage evidence, the berberine, protoberberine, and jatrorrhizine alkaloids present in *Berberis vulgaris* L., *Coptis Chinensis HuangLian*, *Hydrastis canadensis* L., and *Mahonia aquifolium* (Pursh) Nutt have anti‐acne, antioxidant, anti‐inflammatory, and anti‐lipogenic effects [79]. Mishra et al. [88] found the presence of various phytoconstituents, including flavonoids and alkaloids, in *Cassia angustifolia* extract, which in vivo experiments showed the anti‐acne activity [88]. The extract of some herbs, including *Leucaena leuccocephala*, *Nephelium lappaceum* L., *Citrus sinensis* (L.) *osbeck* (orange peel extract), *Curcuma zedoaria*, and *Carica papaya* L., was applied in the preparation of acne face masks because of the presence of ingredients such as tocopherols, saponins, tannins, flavonoids, and alkaloids that may have the potential to show antibacterial properties against *P. acnes* and *S. aureus* [90]. Putri et al. [91] used an extract of rambutan leaves to formulate the peel‐off gel mask. They mentioned that the alkaloids in the extract inhibit the growth of *P. acnes* in an in vitro study [91]. In another survey, a facial gel mask designed with an extract of sweet orange citrus peel showed anti‐acne benefits at concentrations of 10% in an in vitro experiment [92]. Several clinical studies have directly compared natural compounds with conventional acne therapies. A combination of aloe vera gel and tretinoin cream demonstrated superior improvement in both inflammatory and non‐inflammatory lesions compared to tretinoin alone [93]. Similarly, formulations containing *Ocimum gratissimum* essential oil, either alone or combined with aloe vera, showed greater efficacy and faster reduction of inflammatory lesions than clindamycin solution, and were more tolerable than benzoyl peroxide lotion [94, 95]. A topical formulation containing propolis, aloe vera, and tea tree oil outperformed erythromycin cream in reducing acne severity, erythema, and lesion counts [96]. In contrast, a cream containing Casuarina equisetifolia bark extract demonstrated comparable efficacy to benzoyl peroxide, with no significant difference between treatments [97]. Another comparative study found that a formulation containing retinol and rose extract had similar efficacy to adapalene gel but exhibited a better safety profile (Lee et al. [98]). These studies collectively indicate that certain natural agents may offer efficacy comparable to, or in some cases greater than, standard topical therapies, with potential advantages in tolerability.

Optimizing the selection of treatments based on chemical classification and molecular structures plays a crucial role in advancing personalized medicine. By tailoring therapies to the specific chemical properties and structures of a patient's condition, treatment efficacy can be significantly improved. This approach allows for more precise and targeted interventions, reducing side effects and enhancing therapeutic outcomes. A detailed discussion of this topic is provided in the supplementary file.

## 5 Conclusion

AV is still a common dermatological disorder that impairs the quality of life of patients. Although there are numerous treatment options available, it remains difficult for the clinician to choose the best treatment for each patient. This review categorizes popular acne therapies by chemical class and discusses their mechanism of action, clinical efficacy, and potential adverse effects. Of the novel agents discussed, only clascoterone and sarecycline are FDA‐approved, and other compounds have undergone study mainly in phase II designs. Although early phase studies of many patients have produced exciting results, especially for natural compounds, peptide‐based agents, and formulations with nanotechnology, research gaps remain. In particular, human phase III randomized controlled studies of natural and biologically derived compounds must be better designed to determine clinical effectiveness, and safety studies for long‐term use are needed as an example of molecularly designed therapies with nanoparticle delivery systems. Novaul agents are a promising area of research going forward. A better understanding of the molecular pathways that drive the pathogenesis of AV will allow the subsequent development of targeted treatment options that are efficacious and minimize adverse effects. Finally, the continued organization of treatment options by chemical class will aid in a more personalized approach to treatment, ultimately improving acne management and reducing the overall burden of disease.

## Author Contributions

M.T. and S.A. drafted the manuscript and were involved indata collection. R.H. was reponsible acquisition of data. M.M.R., M.B., and E.K.h. were responsible for the study concept and design, supervision, and final editing.

## Ethics Statement

The authors have nothing to report.

## Conflicts of Interest

The authors declare no conflicts of interest.

## Transparency Statement

The corresponding authors Elham Khanniri, Maryam Bayanati, Mohammad Mahboubi‐Rabbani affirms that this manuscript is an honest, accurate, and transparent account of the study being reported; that no important aspects of the study have been omitted; and that any discrepancies from the study as planned (and, if relevant, registered) have been explained.

## Supporting information


**Fig. 1:** Pathogenesis of acne vulgaris.

## Data Availability

Data sharing does not apply to this article as no new data were created or analyzed in this study.
