# Supplementary material for: A Comprehensive Review of Acne Treatments: Unpacking the Chemical Structures and Effective Bioactive Compounds
Source: Health Sci Rep. 2026 Feb 3;9(2):e71803. doi: 10.1002/hsr2.71803 (PMC12865227; doi:10.1002/hsr2.71803)
Supplement: Supplementary file 1 — Fig. 1: Pathogenesis of acne vulgaris. [file HSR2-9-e71803-s001.docx]

**Background on acne pathogenesis**

The development of the disease depends on several important factors (Fig. 1), which include an alteration in microbial flora, abnormal skin keratinization, and inflammation (Rusu et al., 2020). Acne involves a polymicrobial component, including Staphylococcus epidermidis (S. epidermidis), Staphylococcus aureus, and C. acnes. While C. acnes is considered the main pathogen in this process, Staphylococcus species may contribute to inflammatory exacerbation and significant antimicrobial resistance, resulting in treatment failure (Hassanzadeh, Bahmani and Mehrabani, 2008; Nakase et al., 2014; Nishijima et al., 2000). Even though acne is caused as a result of the imbalance among the various phylotypes of C. acnes, this bacterium does not participate in the formation of comedones. Also, the gut microbiome plays a role in the development of acne by its interaction with the skin microbiome. Loss of diversity of C. acnes phylotypes promotes the stimulation of the innate immune system and inflammation (Dréno et al., 2020). More specifically, the proteases, lipase, and hyaluronidase produced by C. acnes can cause inflammation (Firlej et al., 2022). The inflammatory materials like interleukin (IL)-1 and tumor necrosis factor (TNF)-α (Cosio et al., 2021), that cause hyperkeratinization (Del Rosso and Kircik, 2024), are released from keratinocytes by their stimulation with disruption of the epithelium induced by accumulation of sebum. As a result of overproduction and the stickiness of epithelial cells, there is intrafollicular accumulation (Cosio et al., 2021), and because of the excessive proliferation of keratinocytes, comedones are formed (Hazarika, 2021). The main regulators of sebocyte growth and lipid synthesis are androgens, specifically testosterone and dihydrotestosterone, which influence sebum production. High levels of androgens at puberty greatly increase sebum production and, subsequently, the formation of AV. The fatty acids in sebum have comedogenic effects that promote differentiation of keratinocytes and follicular keratinization, which ultimately produce the acne lesion. Sebum may promote C. acnes proliferation as its nutrients derive from sebum (Del Rosso and Kircik, 2024). In general, the number and/or quality of sebum can induce inflammation and subsequently stimulate the mechanisms of immune response (Zouboulis, 2020). The distribution of non-inflammatory lesions— open comedones (blackheads) and closed comedones (whiteheads)—finally presents a pathological state in the development of acne. These lesions are produced through the process of comedogenesis which includes:(1) Hyperproliferation of follicular keratinocytes, as well as abnormal desquamation, resulting in the accumulation of corneocytes (2) Hypersecretion of sebum, precipitated by androgens, that form obstructing plugs in the pilosebaceous ducts with keratinous debris (3) Occult follicular occlusion leading to the formation of microcomedones (subclinical precursors) and creating a hypoxic microenvironment suitable for C. acnes proliferation. Although microcomedones are clinically non-inflammatory, they exist with subclinical inflammation, where keratinocytes will release cellular IL-1α signal, which can activate the nuclear factor-κB (NF-κB) pathways and recruit immune cells. This history of subclinical inflammation takes microcomedones into the threshold of inflammatory lesions once those microcomedones have become colonized with C. acnes (Jeremy et al., 2003; Koreck et al., 2003). Therefore, non-inflammatory lesions should not solely be regarded as cosmetic, rather the initial step that initiates the pathogenic cascade of acne. Insulin-like growth factor 1 (IGF-1) may also be a contributor to the pathogenesis of acne. Evidence is present in the polymorphism of IGF-1 to facilitate the susceptibility to acne by influencing the androgen receptors, and thereby inducing the keratinocytes and sebaceous glands (Hazarika, 2021). The role of diet has also increasingly come to the forefront of the pathogenesis. There may be the potential to provide all necessary components for the formation of either pro-inflammatory or anti-inflammatory sebaceous lipids for acne lesion development through diet. Certain diets may increase IGF-1 (Zouboulis, 2020). The role of diet in acne pathogenesis involves more than modulation of IGF-1, with different diets acting on multiple modes depending on the food types. High-glycemic foods will rapidly increase blood glucose and insulin, which is considered to increase IGF-1 signalling. Increased IGF-1 will stimulate sebocyte proliferation, androgen (testosterone) formation, and also activate lipogenesis via the mTORC1 pathway in sebocytes, with added inflammation. Dairy foods will have the additional contribution of bovine IGF-1, insulinogenic whey proteins, and dietary hormones that are precursors to hormones that convert to dihydrotestosterone (DHT), which would all contribute to the production of testosterone and additionally sebum. Cheeses, particularly, are exposed to bacteria that can carry active DHT into the body, where it is cycled and contributes to new, oily brine. Foods that are spicy or fried contain saturated fats that can act on the TLR2 and TLR4 receptors on sebaceous glands, and can activate TLR2 and TLR4 receptors and allow for the release of IL-1α, TNF-α, and CXCL8. These same saturated fatty acids can also undergo peroxidation in sebum, allowing for the development of free radicals to enhance comedogenic activity and rupture of the follicle by the inflammatory response. Diets that are high in omega-6 fatty acids, such as the vegetable oils, can raise pro-inflammatory leukotriene B4 (LTB4) levels, which can recruit neutrophils and increase follicular rupture. Diets with high levels of all these food kinds exacerbate the pathological pillars of acne: sebaceous lipids production, hyperkeratinization, inflammation, and C. acnes overgrowth (Agak et al., 2014; Juhl et al., 2018; Jung et al., 2014; Melnik, 2015).


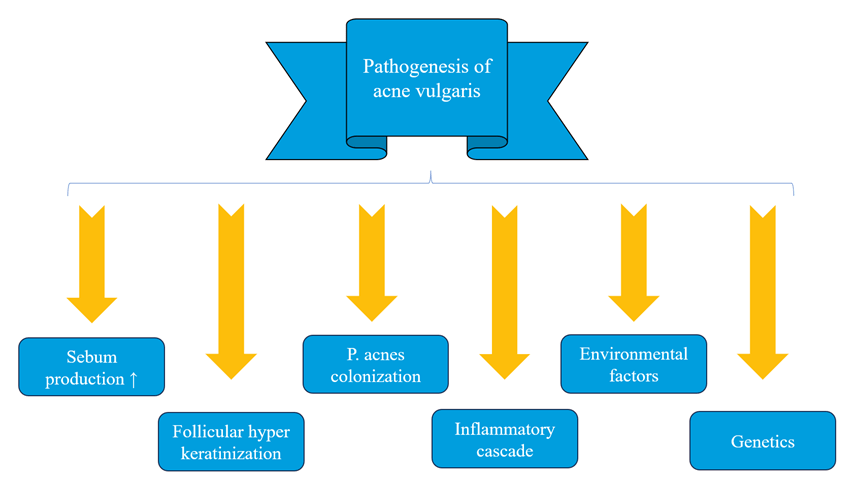


**Fig. 1** Pathogenesis of acne vulgaris

**Optimizing treatment selection based on chemical classification and structures**

A firm understanding of the chemical class and structure of acne therapeutics can significantly enhance treatment selection, offering a promising pathway toward personalized medicine. Classifying acne medications based on their molecular structure enables clinicians to predict better therapeutic efficacy, potential side effects, and drug interactions. Furthermore, deeper insights into mechanisms of action at the molecular level allow for the rational design of new targeted therapies and more refined treatment algorithms based on acne subtype and patient profile (Fouladi, 2012; Rahimi-Madiseh et al., 2017).

Retinoids, such as tretinoin, adapalene (ADP), isotretinoin, tazarotene, alitretinoin, acitretin, bexarotene, and trifarotene, regulate keratinocyte differentiation and proliferation, reduce sebum production, and exhibit anti-inflammatory properties (Kim and Kim, 2024; Rusu et al., 2020). Their detailed chemical structures have been presented earlier in Figures 2 and 3, along with a discussion of their receptor selectivity and generation-based classification. Topical formulations may cause irritation and photosensitivity, while systemic isotretinoin, though highly effective, requires strict monitoring due to its teratogenicity (Bagatin et al., 2020; Vallerand et al., 2017).

Antibacterial agents include several structurally distinct groups. Lincosamides such as Clindamycin and macrolides like erythromycin and Azithromycin inhibit bacterial protein synthesis by binding to the 50S ribosomal subunit (Abdel-Karim et al., 2025; Schwarz et al., 2016). Tetracyclines, including doxycycline, minocycline, and sarecycline, inhibit protein translation by targeting the 30S ribosomal subunit and are widely used systemically for moderate-to-severe acne. Their structures and analogs are illustrated in Figures 4–6 (Armstrong, Hekmatjah and Kircik, 2020; Grada, Ghannoum and Bunick, 2022; Swallow et al., 2022). Dapsone, a sulfone compound effective in inflammatory acne, has a unique structure and mode of action that was previously shown in Figure 7 (Geyfman et al., 2019; Wozel and Blasum, 2014). Sulfur and sodium sulfacetamide are topical sulfonamides with keratolytic and bacteriostatic activity; sulfacetamide’s structure also appears in Figure 7 (G. Alani et al., 2024; Hashem et al., 2021).

BPO, a topical oxidizing agent, destroys C. acnes through the production of free radicals and has mild comedolytic effects. Its chemical structure is provided in Figure 7 (Kim and Kim, 2024; Zhou et al., 2022). Salicylic acid, a β-hydroxy acid, exhibits keratolytic, comedolytic, and anti-inflammatory properties, especially in non-inflammatory lesions. AZA, a saturated dicarboxylic acid, inhibits tyrosinase and 5α-reductase, providing comedolytic and sebostatic effects. Both salicylic acid and AZA structures are also found in Figure 7 (Sauer et al., 2023).

Hormonal therapies have gained importance in the treatment of female patients with acne. Clascoterone, a topical androgen receptor inhibitor, represents a novel non-systemic antiandrogen with a favorable safety profile, and its structure is shown in Figure 8 (Hebert et al., 2020; Sanchez and Keri, 2022). Oral hormonal agents, such as spironolactone and combined oral contraceptives (COCs), are also widely used but are not shown structurally due to their established usage outside the scope of dermatological chemistry. Their mechanisms and therapeutic roles are discussed in the hormonal acne section (Smith et al., 2025; Trivedi, Shinkai and Murase, 2017).

Emerging therapies include a broad array of chemically diverse compounds. CBD, a phytocannabinoid with anti-inflammatory and sebostatic activity, is illustrated in Figure 10 (Oláh et al., 2014). Zileuton and acebilustat, leukotriene-modulating agents, are structurally presented in Figures 11 and 12, respectively (Gollnick, 2015; Zouboulis, 2009). Lupeol, a pentacyclic triterpene with anti-inflammatory and lipogenesis-reducing effects, is also illustrated in Figure 12 (Saleem, 2009). Olumacostat glasaretil, a topical sebum-suppressing agent that inhibits acetyl-CoA carboxylase, and Talarozole, a selective CYP26 inhibitor that increases endogenous retinoic acid, are both included in Figure 12 (Melnik, 2017). Epigallocatechin-3-gallate (EGCG), a green tea-derived polyphenol, acts on multiple acne-related pathways, and its structure is also available in Figure 12 (Collins et al., 2007; Saric, Notay and Sivamani, 2016). Finally, botulinum toxin A (BTX-A), which suppresses sebum production by inhibiting acetylcholine release, is discussed in detail with its three-dimensional structure illustrated in Figure 13 (Birkett et al., 2022).

By consolidating therapeutic options according to their chemical nature and mechanistic profile and by maintaining consistent reference to their chemical structures, this revised chapter ensures greater clarity and coherence. Such an approach supports individualized acne management strategies, enhances clinical outcomes, and minimizes adverse effects and resistance, ultimately aligning with the principles of precision dermatology.

**References**

Abdel-Karim SA, Serry FM, Elmasry EM, et al. Phenotypic and genotypic characteristics of macrolide, lacosamide, and streptogramin resistance in clinically resistant Streptococci and their correlation with reduced biocide susceptibility. BMC Medicine. 2025;23: 281.

Agak GW, Qin M, Nobe J, et al. Propionibacterium acnes Induces an IL-17 Response in Acne Vulgaris that Is Regulated by Vitamin A and Vitamin D. J Invest Dermatol. 2014;134:366-373.

Alani B, Salim K, Saleh A, et al. Sulfonamide derivatives: Synthesis and applications. International Journal of Frontiers in Chemistry and Pharmacy Research. 2024;4: 001–015.

Armstrong AW, Hekmatjah J, Kircik LH. Oral Tetracyclines and Acne: A Systematic Review for Dermatologists. J Drugs Dermatol. 2020;19:s6-s13.

Bagatin E, Costa CS, Rocha M, et al. Consensus on the use of oral isotretinoin in dermatology - Brazilian Society of Dermatology. An Bras Dermatol. 2020; 95:19-38.

Birkett L, Dhar S, Singh P, et al. Botulinum Toxin A in the Management of Acne Vulgaris: Evidence and Recommendations. Aesthet Surg J. 2022;42:Np507-np509.

Collins QF, Liu HY, Pi J, et al. Epigallocatechin-3-gallate (EGCG), a green tea polyphenol, suppresses hepatic gluconeogenesis through 5'-AMP-activated protein kinase. J Biol Chem. 2007; 282:30143-30149.

Cosio T, Di Prete M, Gaziano R, et al. Trifarotene: A Current Review and Perspectives in Dermatology. Biomedicines. 2021;9: 237.

Del Rosso JQ, Kircik L. The primary role of sebum in the pathophysiology of acne vulgaris and its therapeutic relevance in acne management. J Dermatolog Treat. 2024;35:2296855.

Dréno B, Dagnelie MA, Khammari A, et al. The Skin Microbiome: A New Actor in Inflammatory Acne. Am J Clin Dermatol. 2020; 21: 18-24.

Firlej E, Kowalska W, Szymaszek K, et al. The Role of Skin Immune System in Acne. J Clin Med. 2022;11: 1579.

Fouladi RF. Aqueous extract of dried fruit of Berberis vulgaris L. in acne vulgaris, a clinical trial. J Diet Suppl. 2012; 9:253-261.

Geyfman, M., Debabov, D., Poloso, N., & Alvandi, N. (2019). Mechanistic insight into the activity of a sulfone compound dapsone on Propionibacterium (Newly Reclassified as Cutibacterium) Acnes-mediated cytokine production. Exp Dermatol, 28(2), 190-197.

Gollnick HP. From new findings in acne pathogenesis to new approaches in treatment. J Eur Acad Dermatol Venereol. 2015;29: 1-7.

Grada A, Ghannoum MA, Bunick CG. Sarecycline Demonstrates Clinical Effectiveness against Staphylococcal Infections and Inflammatory Dermatoses: Evidence for Improving Antibiotic Stewardship in Dermatology. Antibiotics (Basel). 2022; 11: 722.

Hashem NM, Hosny A, Abdelrahman AA, et al. Antimicrobial activities encountered by sulfur nanoparticles combating Staphylococcal species harboring sccmecA recovered from acne vulgaris. AIMS Microbiol. 2021;7:481-498.

Hassanzadeh P, Bahmani M, Mehrabani D. Bacterial resistance to antibiotics in acne vulgaris: an in vitro study. Indian J Dermatol. 2008;53:122-124.

Hazarika N. Acne vulgaris: new evidence in pathogenesis and future modalities of treatment. J Dermatolog Treat. 2021; 32:277-285.

Hebert A, Thiboutot D, Stein Gold L, et al. Efficacy and Safety of Topical Clascoterone Cream, 1%, for Treatment in Patients With Facial Acne: Two Phase 3 Randomized Clinical Trials. JAMA Dermatol. 2020;156:621-630.

Jeremy AH, Holland DB, Roberts SG, et al. Inflammatory events are involved in acne lesion initiation. J Invest Dermatol. 2003; 121:20-27.

Juhl CR, Bergholdt HKM, Miller IM, et al. Dairy Intake and Acne Vulgaris: A Systematic Review and Meta-Analysis of 78,529 Children, Adolescents, and Young Adults. Nutrients. 2018;10: 1049.

Jung JY, Kwon HH, Hong JS, et al. Effect of dietary supplementation with omega-3 fatty acid and gamma-linolenic acid on acne vulgaris: a randomised, double-blind, controlled trial. Acta Derm Venereol. 2014; 94:521-525.

Kim HJ, Kim YH. Exploring Acne Treatments: From Pathophysiological Mechanisms to Emerging Therapies. Int J Mol Sci. 2024; 25: 5302.

Koreck A, Pivarcsi A, Dobozy A, et al. The role of innate immunity in the pathogenesis of acne. Dermatology. 2003; 206:96-105.

Melnik BC. Linking diet to acne metabolomics, inflammation, and comedogenesis: an update. Clin Cosmet Investig Dermatol. 2015;8:371-388.

Melnik BC. Olumacostat Glasaretil, a Promising Topical Sebum-Suppressing Agent that Affects All Major Pathogenic Factors of Acne Vulgaris. J Invest Dermatol. 2017; 137:1405-1408.

Nakase K, Nakaminami H, Takenaka Y, et al. Relationship between the severity of acne vulgaris and antimicrobial resistance of bacteria isolated from acne lesions in a hospital in Japan. J Med Microbiol. 2014; 63:721-728.

Nishijima S, Kurokawa I, Katoh N, et al. The bacteriology of acne vulgaris and antimicrobial susceptibility of Propionibacterium acnes and Staphylococcus epidermidis isolated from acne lesions. J Dermatol. 2000; 27:318-323.

Oláh A, Tóth BI, Borbíró I, et al. Cannabidiol exerts sebostatic and antiinflammatory effects on human sebocytes. J Clin Invest. 2014;124:3713-3724.

Rahimi-Madiseh M, Lorigoini Z, Zamani-Gharaghoshi H, Rafieian-Kopaei M. Berberis vulgaris: specifications and traditional uses. Iran J Basic Med Sci. 2017; 20: 569-587.

Rusu A, Tanase C, Pascu G A, et al. Recent Advances Regarding the Therapeutic Potential of Adapalene. Pharmaceuticals (Basel). 2020;13:217.

Saleem M. Lupeol, a novel anti-inflammatory and anti-cancer dietary triterpene. Cancer Lett. 2009;285:109-115.

Sanchez C, Keri J. Androgen Receptor Inhibitors in the Treatment of Acne Vulgaris: Efficacy and Safety Profiles of Clascoterone 1% Cream. Clin Cosmet Investig Dermatol. 2022;15,1357-1366.

Saric S, Notay M, Sivamani RK. Green Tea and Other Tea Polyphenols: Effects on Sebum Production and Acne Vulgaris. Antioxidants (Basel). 2016; 6:2.

Sauer N, Oślizło M, Brzostek M, et al. The multiple uses of azelaic acid in dermatology: mechanism of action, preparations, and potential therapeutic applications. Postepy Dermatol Alergol. 2023; 40:716-724.

Schwarz S, Shen J, Kadlec K, et al. Lincosamides, Streptogramins, Phenicols, and Pleuromutilins: Mode of Action and Mechanisms of Resistance. Cold Spring Harb Perspect Med. 2016;6.

Smith C A, Gosnell E, Karatas TB, et al. Hormonal Therapies for Acne: A Comprehensive Update for Dermatologists. Dermatol Ther (Heidelb). 2025;15:45-59.

Swallow M A, Fan R, Cohen JM, Bunick CG. Antibiotic Resistance Risk with Oral Tetracycline Treatment of Acne Vulgaris. Antibiotics (Basel). 2022;11:1032.

Trivedi MK, Shinkai K, Murase JE. A Review of hormone-based therapies to treat adult acne vulgaris in women. Int J Womens Dermatol. 2017; 3: 44-52.

Vallerand I, Lewinson R, Farris M, et al. Efficacy and Adverse Events of Oral Isotretinoin for Acne: A Systematic Review. Br J Dermatol. 2017; 178:76–85.

Wozel G, Blasum C. Dapsone in dermatology and beyond. Arch Dermatol Res. 2014;306;103-124.

Zhou L, Chen L, Liu X, et al. The influence of benzoyl peroxide on skin microbiota and the epidermal barrier for acne vulgaris. Dermatol Ther. 2022; 35:e15288.

Zouboulis CC. Zileuton, a new efficient and safe systemic anti-acne drug. Dermatoendocrinol. 2009;1:188-192.

Zouboulis CC. Endocrinology and immunology of acne: Two sides of the same coin. Exp Dermatol. 2020;29:840-859.
